# Brown Algae *Ecklonia cava* Extract Modulates Adipogenesis and Browning in 3T3-L1 Preadipocytes through HO-1/Nrf2 Signaling

**DOI:** 10.3390/md22080330

**Published:** 2024-07-23

**Authors:** Indyaswan T. Suryaningtyas, Dae-Sung Lee, Jae-Young Je

**Affiliations:** 1 Department of Food and Nutrition, Pukyong National University, Busan 48513, Republic of Korea; indy001@brin.go.id; 2 Research Center for Food Technology and Processing, National Research and Innovation Agency, Yogyakarta 55861, Indonesia; 3 National Marine Biodiversity Institute of Korea (MABIK), Seochun 33662, Republic of Korea; daesung@mabik.re.kr; 4 Major of Human Bioconvergence, Division of Smart Healthcare, Pukyong National University, Busan 48513, Republic of Korea

**Keywords:** *Ecklonia cava*, adipogenesis, adipose browning, HO-1/Nrf2

## Abstract

This study explores the anti-obesity effects of the ethyl acetate extract of *Ecklonia cava* (EC-ETAC) on 3T3-L1 preadipocytes, focusing on its impact on adipogenesis, lipolysis, and adipose browning via the HO-1/Nrf2 pathway. Western blot analysis revealed that EC-ETAC significantly inhibited adipogenic transcription factors (PPARγ, C/EBPα, SREBP-1) and lipogenesis-related proteins (FAS, LPL). Concurrently, EC-ETAC enhanced lipolytic markers (p-AMPK, p-HSL) and adipose browning-related proteins (UCP-1, PGC-1α), indicating its role in promoting lipolysis and adipose browning. The inhibition of HO-1 by zinc protoporphyrin (ZnPP) significantly reversed these effects, underscoring the critical role of HO-1 in mediating the anti-obesity properties of EC-ETAC. Additionally, fluorescence measurements and Oil Red O staining confirmed the reduction of lipid accumulation and oxidative stress upon EC-ETAC treatment. These findings suggest that EC-ETAC exerts its anti-obesity effects by modulating the HO-1/Nrf2 pathway, which is crucial for regulating adipogenesis, lipolysis, and adipose browning. This study highlights the potential of EC-ETAC as a natural therapeutic agent for obesity management and supports further research into its clinical applications. By targeting the HO-1/Nrf2 pathway, EC-ETAC could offer a novel approach to enhancing energy expenditure and reducing fat mass, thereby improving metabolic health.

## 1. Introduction

Obesity results from a persistent energy imbalance, where caloric intake surpasses expenditure. This surplus is stored as triglycerides in white adipose tissue (WAT), increasing both the number and size of adipocytes [1]. The process of adipogenesis, where preadipocytes become mature adipocytes, is tightly controlled by transcription factors like peroxisome proliferator-activated receptor gamma (PPARγ) and CCAAT/enhancer-binding proteins (C/EBPs) [2]. Meanwhile, brown adipose tissue (BAT) represents another type of adipose tissue, characterized by the presence of numerous mitochondria rich in uncoupling protein 1 (UCP-1). UCP-1 plays a crucial role in this process by uncoupling oxidative phosphorylation, leading to the generation of heat instead of ATP [3]. In addition to inhibiting adipogenesis, enhancing the function and number of BAT can effectively combat obesity [4].

Clusters of thermogenic adipocytes, referred to as beige or brown-like adipose tissue, can develop within WAT in response to various stimuli, such as cold exposure or specific hormonal signals [3,5]. Beige adipocytes share several essential characteristics with those found in BAT. They contain multilocular lipid droplets, exhibit high mitochondrial density, and express specific brown fat genes, such as UCP1 and PPARγ coactivator 1-alpha (PGC-1α), which are vital for their thermogenic function [6]. Despite both having thermogenic capabilities, brown and beige adipocytes are distinct cell types. Brown adipocytes, derived from a myogenic lineage, are found in specific depots, while beige adipocytes form within WAT in response to stimuli and originate from a different lineage [3].

The capacity to induce the conversion of WAT to a brown-like phenotype, known as browning, holds substantial promise for enhancing metabolic health. Stimulating this process represents a potential therapeutic approach against obesity and metabolic disorders by boosting energy expenditure and enhancing glucose metabolism. This strategy targets adipose tissue plasticity to promote a healthier metabolic profile and mitigate the adverse effects associated with excess adiposity [4,7].

Recent research has highlighted the role of heme oxygenase-1 (HO-1) in the modulation of obesity-related pathways [8,9,10]. HO-1, a stress-responsive enzyme, helps protect cells from oxidative damage by breaking down heme into biliverdin, carbon monoxide, and free iron. Up-regulation of HO-1 has been demonstrated to mitigate fat deposition and inhibit the differentiation and hypertrophy of adipocytes. HO-1 also interacts with AMP-activated protein kinase (AMPK), a crucial energy regulator, indicating its role in metabolic regulation [11]. A study found that inducing HO-1 is associated with the browning of perivascular adipose tissue (PVAT) in mice fed a high-fat diet [12,13,14]. These findings indicate that HO-1 may play a critical role in influencing the phenotype of adipocytes, including their browning characteristics.

Brown seaweed *Ecklonia cava* (EC), has garnered interest for its potential as a natural treatment for obesity, offering an alternative to pharmaceuticals with fewer side effects [15,16]. It contains bioactive compounds like polyphenols (phlorotannin), fucoidans, and carotenoids, known for their antioxidant, anti-inflammatory, and metabolic-regulating effects [16,17,18]. These properties may influence adipogenesis and adipocyte phenotype. Yet, the potential of EC in improving obesity through the induction of adipose tissue browning remains poorly understood. This study investigates how EC extract can prevent adipogenesis and promote the browning of 3T3-L1 preadipocytes. It also examines the molecular mechanisms, particularly the HO-1/nuclear factor erythroid 2-related factor 2 (Nrf2) pathway, which regulates adipocyte metabolism and thermogenesis.

## 2. Results

### 2.1. Screening of Anti-Adipogenic Activity from EC Extracts

**Figure 1:**
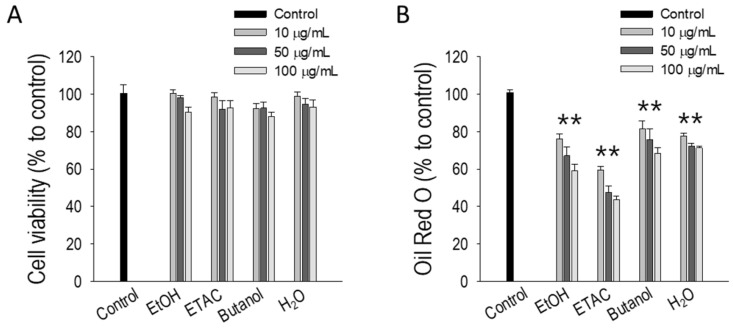
Cytotoxicity and anti-adipogenic screening of EC samples. (**A**) Cell viability of 3T3-L1 preadipocytes treated with EC extracted with various solvents (ethanol, ethyl acetate, butanol, and water in different concentrations (0, 10, 50, and 100 µg/mL)) evaluated using the MTT assay. (**B**) The anti-obesity activity of EC extracts on 3T3-L1 preadipocyte differentiation, measured by Oil Red O assay after 8 days under adipogenic medium. Samples were tested in different concentrations with untreated fully differentiated cells as control. Quantification of stained lipid droplets was performed using a microplate reader at 520 nm absorbance. Values show mean ± STDEV (*n* = 3). The data is expressed as mean ± SD, ** *p* < 0.01, compared to the control group (untreated cells).

A screening test was conducted using EC extracts prepared with various solvents: ethanol (ETOH), ethyl acetate (ETAC), butanol, and water (H_2_O), yielding 21.7%, 17.2%, 7.2%, and 51.3%, respectively. Initial cell viability tests confirmed that none of the extracts were toxic to 3T3-L1 preadipocytes (Figure 1A). Subsequently, the extracts were tested at three different concentrations (10, 50, and 100 µg/mL) in an adipogenic medium for 8 days to allow the preadipocytes to fully differentiate and form lipid droplets. Following Oil Red O staining to assess lipid accumulation, all extracts demonstrated significant anti-adipogenic effects (*p* < 0.05). However, the ethanol (EC-ETOH) and ethyl acetate (EC-ETAC) extracts exhibited the highest inhibition of lipid accumulation (*p* < 0.01) (Figure 1B). Based on these findings, EC-ETAC was selected for further experiments due to its superior efficacy in reducing lipid accumulation in 3T3-L1 preadipocytes.

### 2.2. Chemical Composition of EC-ETAC

The ultra-high-resolution Q-TOF LC MS/MS analysis of the EC-ETAC identified a variety of compounds, characterized by their molecular weight (*m*/*z*), mass error (ppm), retention time (RT) (min), and percent area (%), as presented in Table 1. The peak area of each compound indicates its relative abundance within the extract, with the area percentage calculated to reflect its proportion of the total extract composition. Among the identified compounds, dieckol emerged as the most abundant, comprising 21.49% of the total extract area (molecular weight 741.0727, RT 12.27 min). This was followed by dibenzodioxin-fucodiphloroethol, which accounted for 11.33% of the total area (molecular weight 743.0882, RT 10.32 min), and 8,8′-bieckol, representing 10.20% of the extract (molecular weight 741.0726, RT 9.29 min). Other notable compounds included 2-O-(2,4,6-trihydroxyphenyl)-6,6′-bieckol at 8.53% (molecular weight 865.0885, rt 9.38 min) and 7-phloroeckol at 7.48% (molecular weight 495.0570, RT 8.53 min). These dominant compounds, alongside others identified in the extract, were confirmed through spectral data comparison with known reference standards and databases such as METLIN and MassBank. This rigorous identification process ensures the reliability of the results. The presence of these compounds in significant proportions highlights their potential contributions to the overall bioactivity of the EC-ETAC extract.

### 2.3. Lipid Accumulation in 3T3-L1 Preadipocytes after EC-ETAC Treatment

**Figure 2:**
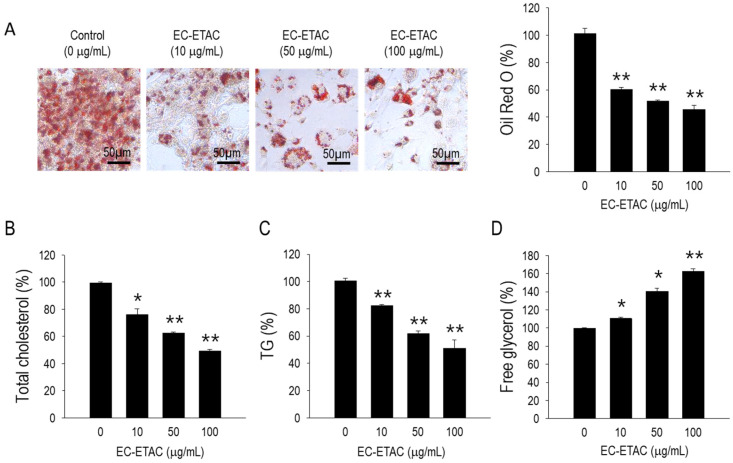
Anti-adipogenic effects of EC-ETAC on 3T3-L1 preadipocyte cells. The cells were treated with EC-ETAC in 0, 10, 50, and 100 µg/mL concentrations and cultured under adipogenic medium. (**A**) Oil red O image observed using light microscope with 40× magnification, and quantified using microplate reader in 520 nm absorbance, (**B**) total cholesterol, (**C**) total triglyceride, (**D**) free glycerol of 3T3-L1 preadipocyte treated with EC-ETAC, measured using assay kit. The data is expressed as mean ± SD (*n* = 3), * *p* < 0.05, and ** *p* < 0.01, compared to the control group (untreated cells).

The effects of the EC-ETAC on lipid accumulation in fully differentiated 3T3-L1 adipocytes were assessed over an 8-day treatment period. Significant inhibition of lipid accumulation was observed at all tested concentrations of EC-ETAC (*p* < 0.05), but in the most pronounced effect at 100 µg/mL (*p* < 0.01) (Figure 2A). Quantitatively, the average inhibition of lipid accumulation at 100 µg/mL was 45.63 ± 3.10%. Additionally, measurements of total cholesterol and triglycerides revealed a significant reduction compared to the control group (Figure 2B,C). In contrast, levels of free glycerol increased significantly, with averages reaching 162 ± 2.83% compared to the control (Figure 2D). These findings indicate that EC-ETAC not only inhibits lipid accumulation but also influences lipid metabolism, leading to decreased cholesterol and triglyceride levels and increased free glycerol levels in fully differentiated 3T3-L1 adipocytes.

### 2.4. EC-ETAC Inhibits Adipogenesis and Induces Adipose Browning

**Figure 3:**
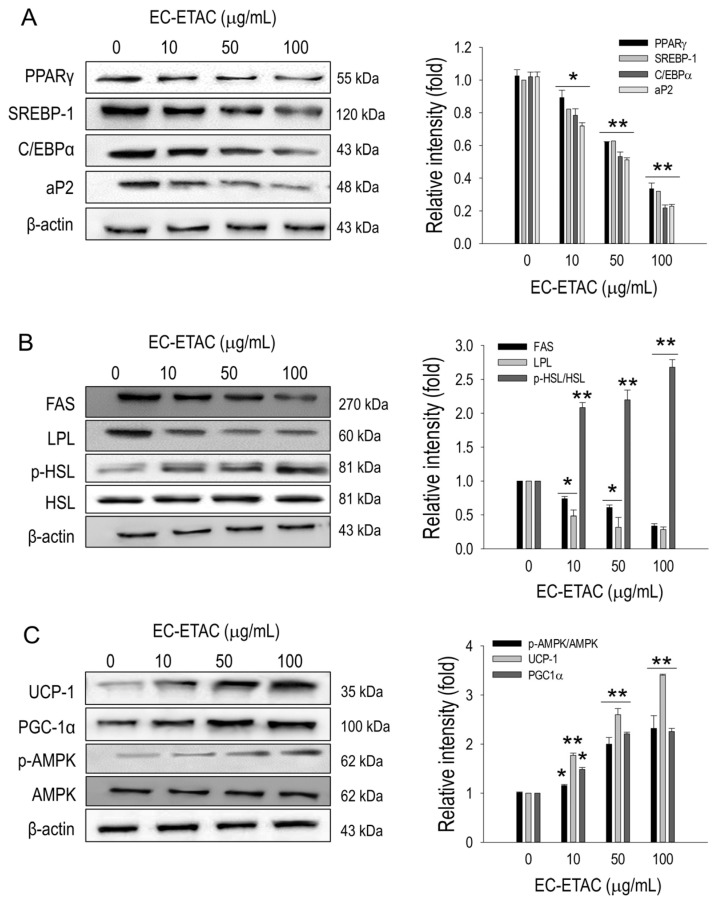
Effects of EC-ETAC on protein expression related to adipogenesis, lipid metabolism, and adipose browning in 3T3-L1 preadipocytes. The 3T3-L1 preadipocytes were treated with varying concentrations of EC-ETAC (0, 10, 50, and 100 µg/mL) and cultured in adipogenic medium. Protein expression levels were evaluated using Western blot analysis, and quantification was performed with ImageJ software (version v1.54g). (**A**) Adipogenic transcription factors, (**B**) lipid metabolism-related proteins, and (**C**) adipose browning-related proteins and phosphorylated AMPK. Following 8 days of differentiation, proteins were collected, quantified using a BCA assay, and normalized. Data are expressed as mean ± SD (*n* = 3). Statistical significance is indicated as * *p* < 0.05 and ** *p* < 0.01, compared to the control group (untreated cell).

The observed reduction in lipid accumulation is likely due to the modulation of adipogenesis, prompting an evaluation of adipogenic transcription factors. Western blot analysis and subsequent quantification revealed that PPARγ, sterol regulatory element-binding protein 1 (SREBP-1), C/EBPα, and adipocyte fatty-acid-binding protein 2 (aP2) expression were significantly inhibited by EC-ETAC treatment at all concentrations (*p* < 0.01) (Figure 3A). Enzymes associated with lipogenesis were also assessed, revealing that the expression of fatty acid synthase (FAS) and lipoprotein lipase (LPL) was markedly inhibited (*p* < 0.01) (Figure 3B). The elevated levels of free glycerol might be the effect of increased lipolytic activity. Therefore, proteins involved in the lipolysis mechanism were evaluated, revealing a significant increase in the expression of phosphorylated AMPK (p-AMPK) and phosphorylated hormone-sensitive lipase (p-HSL) (*p* < 0.01) (Figure 3B). Additionally, proteins indicative of adipose browning, such as UCP1 and PGC-1α, were evaluated. Both proteins exhibited significant upregulation compared to the control group (*p* < 0.01), suggesting an induction of adipose browning activity in 3T3-L1 preadipocytes treated with EC-ETAC (Figure 3C).

### 2.5. HO-1/Nrf2 Pathway Activation

**Figure 4:**
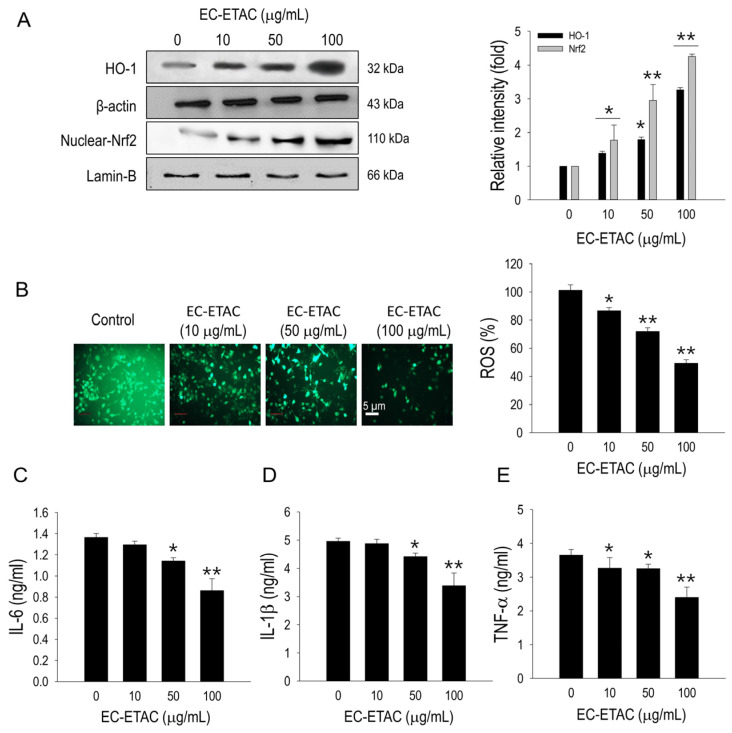
EC-ETAC induces activation of HO-1/Nrf2. (**A**) Western blot image and quantification using ImageJ software of HO-1 and nuclear Nrf2 of 3T3-L1 preadipocytes. After the reaching differentiation state, the cultured cell groups were collected and proteins were measured with BCA assay and normalized. The cells were treated with different concentrations of EC-ETAC in 0, 10, 50, and 100 µg/mL and cultured under adipogenic medium. (**B**) Representative fluorescence image showing intracellular ROS levels generated by adipogenesis process. Cells were cultured until fully differentiated before being stained with DCFH-DA. ROS levels were quantified using a microplate reader, as measured by fluorescence intensity at 485 and 528 nm excitation and emission wavelengths, respectively. Effects of EC-ETAC on the levels of pro-inflammatory cytokines IL-6 (**C**), IL-1β (**D**), and TNF-α (**E**) in 3T3-L1 cells were determined. The 3T3-L1 cells were plated at a density of 4.0 × 10^4^ cells/mL in a 12-well plate. After 2 days of confluency, the media were replaced with differentiation media to induce differentiation into adipocytes. Samples were added every 2 days during adipogenesis process in 10, 50, and 100 μg/mL concentration. Cytokines IL-6, IL-1β, and TNF-α levels in the culture medium were detected by commercial ELISA kit by Biolegend (San Diego, CA, USA) according to the manufacturer’s instructions. The data is expressed as mean ± SD (*n* = 3), * *p* < 0.05, and ** *p* < 0.01, compared to the untreated cells.

The activation of HO-1 by EC-ETAC was assessed using Western blot analysis, which revealed a significant upregulation compared to the control (Figure 4A). Since HO-1 activation is linked to the translocation of Nrf2 into the nucleus, the expression of Nrf2 in the nucleus was also evaluated. A significant increase in nuclear Nrf2 protein levels was observed following EC-ETAC treatment (Figure 4A). The modulation of HO-1 and Nrf2 by EC-ETAC is closely related to the regulation of reactive oxygen species (ROS) generation and inflammation, which are critical during adipogenesis. Strong intensity of fluorescence measurement indicates high ROS levels in the control group. However, the intensity diminished progressively following the increasing concentrations of EC-ETAC. Quantitative analysis confirmed a significant inhibition of ROS generation following EC-ETAC treatment (Figure 4B). Lipid over-accumulation might induce inflammation. Therefore, the levels of pro-inflammatory cytokines were measured to evaluate the impact of EC-ETAC treatment on reducing inflammation. The results demonstrated a significant decrease in the concentration of pro-inflammatory cytokines in treatments with higher EC-ETAC concentrations (*p* < 0.05) (Figure 4C–E). This indicates that EC-ETAC effectively reduces inflammation associated with lipid accumulation by modulating the Nrf2-HO-1 pathway and inhibiting ROS production.

### 2.6. HO-1 Inhibition Effect

**Figure 5:**
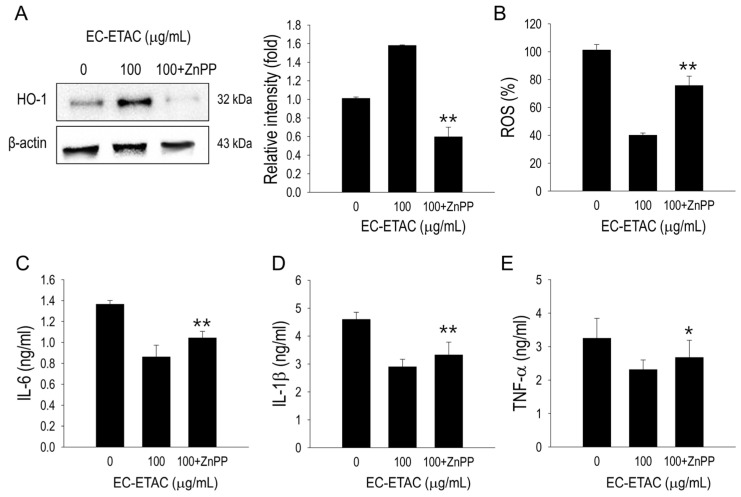
The effect of HO-1 inhibition in ROS generation and inflammation. (**A**) Western blot image and quantification of HO-1 concentration with and without the addition of 5 μM ZnPP in 3T3-L1 preadipocytes treated with EC-ETAC at 100 μM concentration. (**B**) ROS level quantification measured by fluorescence at 485 and 528 nm excitation and emission wavelengths, respectively. The effects of ZnPP addition on the levels of pro-inflammatory cytokines IL-6 (**C**), IL-1β (**D**), and TNF-α (**E**) in 3T3-L1 preadipocytes after EC-ETAC treatment at 100 μM concentration as determined by ELISA Kit. Control group is 3T3-L1 preadipocytes without peptide addition. All values are presented as means ± SD (*n* = 3). * *p* < 0.05 and ** *p* < 0.01 compared to group with sample only (no ZnPP addition).

To understand the role of HO-1 in the anti-obesity mechanism, we added 5 μM zinc protoporphyrin (ZnPP) as an HO-1 inhibitor and evaluated the effects. As shown in Figure 5A, HO-1, which was initially upregulated by EC-ETAC, was successfully inhibited by ZnPP treatment. The reduction in HO-1 concentration also affected the ROS generated during the adipogenesis process, as indicated by a significant increase in fluorescence intensity (*p* < 0.01) (Figure 5B). Furthermore, the levels of pro-inflammatory cytokines increased significantly compared to those of the 100 µg/mL EC-ETAC-treated group (*p* < 0.05) (Figure 5C–E).

### 2.7. HO-1 Inhibition Effect on Lipid Accumulation

**Figure 6:**
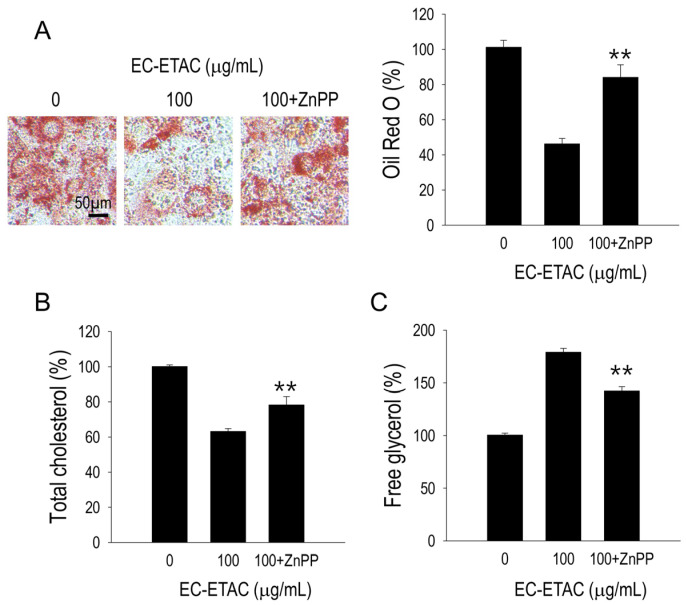
EC-ETAC anti-obesity effects were evaluated on 3T3-L1 preadipocyte cells with and without ZnPP (5 µM) as HO-1 inhibitor. The 3T3-L1 preadipocytes were treated with EC-ETAC at 100 µg/mL and cultured under adipogenic medium with or without ZnPP addition at every medium change. Fully differentiated cells with no treatment were used as control. (**A**) After reaching the differentiation state, cells were then stained with Oil Red O to visualize lipid droplets and observe using light microscope with 40× magnification; quantifications were performed using microplate reader in 520 nm absorbance. The measurement of total cholesterol (**B**) and free glycerol (**C**) using assay kit. The data is expressed as mean ± SD (*n* = 3), ** *p* < 0.01, compared to group with sample only (no ZnPP addition).

The inhibitory effect on lipid accumulation observed with EC-ETAC treatment was diminished after ZnPP pre-treatment. Visually, lipid droplets reappeared when HO-1 was inhibited, and quantitatively, there was a significant increase in Oil Red O absorbance (*p* < 0.01), indicating enhanced lipid accumulation (Figure 6A). Cholesterol levels showed a considerable rise in the absence of HO-1 (*p* < 0.01) (Figure 6B). Furthermore, the concentration of free glycerol decreased significantly (*p* < 0.01) when HO-1 was inhibited (Figure 6C).

**Figure 7:**
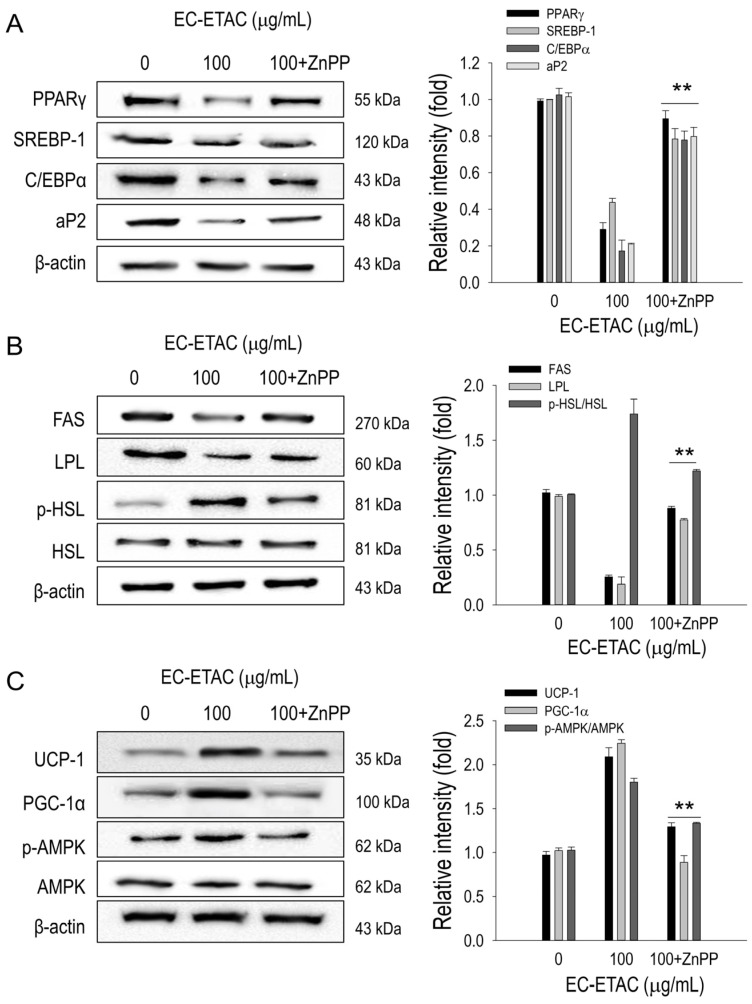
Effects of ZnPP (5 µM) on anti-obesity activity of EC-ETAC. The 3T3-L1 preadipocytes were treated with EC-ETAC in 100 µg/mL, and after 8 days cultured under adipogenic medium with or without ZnPP addition. Proteins were collected, quantified using a BCA assay, and normalized. Fully differentiated cells with no treatment were used as control. Western blot image and quantification using ImageJ software of (**A**) adipogenesis transcription factors, (**B**) lipid metabolism-related protein, and (**C**) adipose browning-related protein and phosphorylated AMPK. The data is expressed as mean ± SD (*n* = 3), ** *p* < 0.01, compared to group with sample only (no ZnPP addition).

To further understand the underlying mechanisms, we investigated the involved pathways. ZnPP suppression of HO-1 led to increased levels of adipogenic transcription factors, which had been previously inhibited by EC-ETAC (Figure 7A). In comparison to the treatment with EC-ETAC alone, the reappearance of lipogenesis-related proteins such as FAS and LPL was seen to be statistically significant (*p* < 0.01) (Figure 7B). The activation of lipolytic markers, p-HSL, showed a considerable decrease (Figure 7B). Furthermore, the activity of browning was diminished, as indicated by the reduced expression of UCP-1 and PGC-1α, which were previously stimulated by EC-ETAC (Figure 7C). EC-ETAC treatment was also shown to induce activation of AMPK but then, after HO-1 inhibition, the concentration of activated AMPK was reduced (Figure 7C) The results emphasize the crucial function of HO-1 in facilitating the anti-adipogenic, lipolytic, and browning effects of EC-ETAC.

## 3. Discussion

The main challenge in reducing obesity lies in the body’s allostatic regulatory mechanisms that control energy balance. Despite the apparent simplicity of addressing energy intake and expenditure, practical clinical implementation is far from straightforward. Therefore, innovative therapeutic strategies that specifically target adipose tissue function could offer promising avenues for future obesity treatment. Marine algae have become a valuable source of natural products, known for their safety and ease of accessibility. Among these are polyphenols, fucoidan, fucoxanthin, and fucosterol, all of which contain active compounds that have shown promising anti-obesity properties and prevent related metabolic disorders [17,19,20,21].

While numerous studies have explored the anti-obesity properties of EC extracts from various fractions [15,16,18], the current research specifically focuses on the involvement of the HO-1 pathway in the anti-obesity mechanism that have not been reported in the context of EC extracts. Previous research has shown that brown seaweed can inhibit adipogenesis and enhance lipolysis through various pathways, such as the AMPK pathway [20], AKT signaling pathway [22], MAPK pathway [23], and PI3K/Akt and ERK-dependent FoxO signaling pathway [24]. However, information regarding the role of HO-1 in these anti-obesity mechanisms is still lacking.

HO-1 is an antioxidant enzyme that is activated at the transcriptional level, offering cellular protection during stress [25]. Recently, innovative therapeutic strategies targeting HO-1 and biliverdin have garnered increasing interest for their potential to improve obesity, adipocyte dysfunction, and related metabolic syndrome. This interest is supported by studies showing a strong correlation between HO-1 activity and the regulation of obesity [9,26,27,28].

Regulation of adipogenesis is a critical pathway for controlling or reversing obesity. PPARγ, as an essential regulator of adipogenesis, has been a primary target in the development of numerous anti-obesity drugs [9,23,29]. While PPARα and PPARδ also play significant roles in lipid metabolism and energy homeostasis, their primary functions are associated with fatty acid oxidation and metabolic regulation in other tissues, such as the liver and muscle [30,31]. Our study focused exclusively on adipocytes, thus we concentrated on PPARγ. Certain natural products can directly regulate PPARγ, while others can modulate its upstream regulators. C/EBPα, for instance, activates the expression of several downstream target genes such as PPARγ, LPL, SREBP-1, and aP2 [29]. In this study, EC-ETAC was found to inhibit PPARγ and other proteins involved in adipogenesis, lipogenesis, and lipolysis enhancement. However, the specific mechanisms by which many natural products regulate PPARγ and its upstream regulators remain unclear. We hypothesize that HO-1 also acts as an upstream regulator for PPARγ, a property mediated by EC-ETAC. The effects observed are likely mediated indirectly through the HO-1/Nrf2 pathway. Activation of HO-1/Nrf2 by EC-ETAC influences PPAR activity by modulating cellular stress responses, inflammation, and oxidative stress, which in turn affect PPAR signaling and function.

Adipogenic medium has been shown to suppress the expression of HO-1 [11,26]. This suppression reduces the cell’s ability to counteract oxidative stress and maintain metabolic balance, potentially contributing to the dysregulation of adipocyte function and promoting the development of obesity-related metabolic disorders. EC-ETAC has been found to induce HO-1/Nrf2 activation, followed by improvement in ROS generation and inflammation as a result of the adipogenesis process. These findings also support the role of HO-1 in mitigating obesity and regulating oxidative stress and inflammation. This is evidenced by the fact that inhibition of HO-1 by ZnPP diminishes the beneficial effects of EC-ETAC on lipid accumulation and metabolism. The activation of the HO-1/Nrf2 pathway by EC-ETAC was also linked to increased levels of p-AMPK and p-HSL, enhancing lipolysis and reducing lipid accumulation within adipocytes.

EC was extracted using various solvents, with the ethyl acetate fraction showing the highest lipid accumulation inhibition activity. Ethyl acetate is particularly effective in extracting phlorotannins, which are abundant in brown seaweeds like EC. To accurately identify the bioactive compounds in the EC-ETAC extract, we utilized high-resolution mass spectrometry (Q-TOF LC-MS/MS) due to its exceptional sensitivity, mass accuracy, and suitability for non-volatile and thermally labile compounds. Compound identification analysis revealed that dieckol dominates the EC-ETAC composition at 21.49%. Dieckol has been previously reported to inhibit adipogenic transcription factors through AMPK activation in 3T3-L1 preadipocytes [32,33,34]. Additionally, 8,8′-bieckol, which constitutes 10.20% of the extract, has been shown to reduce obesity in 3T3-L1 preadipocytes and HFD mice [17,33]. Furthermore, 8,8′-Bieckol has also been reported to have positive effects on glucose levels and hypertension [35].

Promoting the browning of WAT is another promising strategy for obesity management and its related metabolic diseases by enhancing energy expenditure and reducing fat mass [36]. Recent research has focused on transcriptional modulators involved in the browning of white fat depots to better understand how this process can be leveraged to combat obesity. Natural compounds such as resveratrol, quercetin, rutin, and epicatechin have been shown to induce WAT browning by activating the AMPK pathway and promoting mitochondrial biogenesis [14,37]. Similarly, our findings demonstrate that EC-ETAC significantly upregulates the expression of UCP-1 and PGC-1α, key markers of adipose browning. This suggests that EC-ETAC not only inhibits adipogenesis but also promotes the conversion of WAT to BAT, thereby increasing thermogenic capacity and energy expenditure.

**Figure 8:**
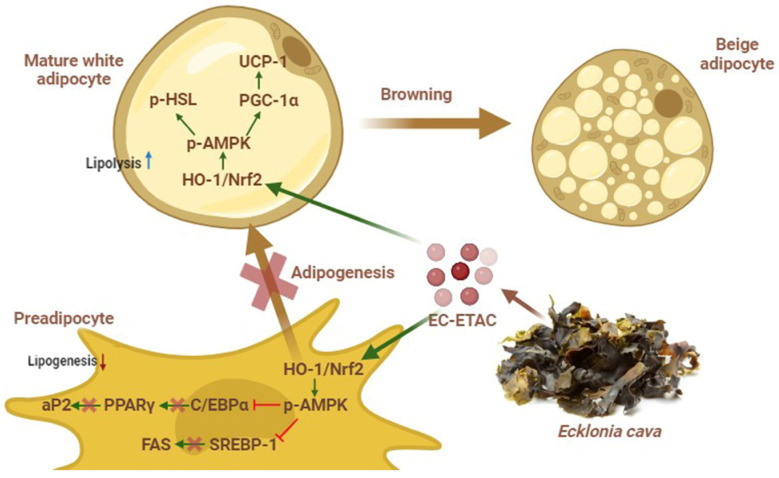
Schematic diagram illustrating the anti-obesity effects of EC-ETAC. EC-ETAC inhibits adipogenesis by downregulating key transcription factors (PPARγ, C/EBPα, SREBP-1), suppresses lipogenesis, enhances lipolysis through upregulation of p-AMPK and p-HSL, and induces browning of white adipose tissue by promoting the expression of browning-related proteins (UCP-1, PGC-1α).

PGC-1α acts as a coactivator for PPARγ, enhancing its transcriptional activity. This coactivation is crucial for the induction of genes involved in mitochondrial biogenesis and oxidative metabolism. PGC-1α not only promotes the browning of WAT by upregulating UCP-1 and other thermogenic genes but also enhances the overall oxidative capacity of cells [3]. Through the activation of PPARγ by PGC-1α, there is a coordinated regulation of lipid metabolism, energy homeostasis, and thermogenesis, making this interaction a pivotal point in the management of metabolic diseases such as obesity and diabetes [6,7]. Interestingly, EC-ETAC shown to upregulates PGC-1α while simultaneously inhibiting PPARγ expression. It is highly likely for a compound with anti-adipogenic effects, which inhibit the formation of white adipocytes, to simultaneously promote the browning of WAT, leading to the development of beige or brite adipocytes [38,39,40]. This dual capability exists because the molecular pathways governing WAT adipogenesis and browning are intricately interconnected and can be modulated by a variety of signaling molecules and transcription factors [6,41]. Figure 8 illustrates that one key mechanism by which EC-ETAC exerts its action involves the upregulation of HO-1, which subsequently activates AMPK.

Recent studies have shown that PGC-1α upregulates HO-1 expression, suggesting a feedback loop where enhanced mitochondrial function and reduced oxidative stress promote further metabolic efficiency [42]. Additionally, the activation of HO-1 has been linked to increased PGC-1α activity, further promoting the browning of WAT and enhancing overall energy expenditure [43,44,45]. This interplay between HO-1 and PGC-1α underscores their potential as therapeutic targets for metabolic disorders, offering a dual approach to reducing oxidative stress and improving metabolic health.

Although 3T3-L1 preadipocytes are commonly used to represent WAT, evidence suggests they can exhibit brown-like characteristics under certain conditions. Exposure to hormones, thermogenic conditions, or PPARγ agonists is known to induce a browning phenotype in 3T3-L1 preadipocyte, resulting in the expression of brown fat markers such as UCP1 and PGC-1α, despite being at lower levels than those found in primary brown or beige adipocytes [3]. While 3T3-L1 cells are valuable for studying adipogenesis and various aspects of adipocyte biology, they do not fully replicate the complexity of primary brown or beige adipocytes [37]. Therefore, findings from 3T3-L1 cell studies should be validated using primary cell cultures or in vivo models.

## 4. Materials and Methods

### 4.1. Materials

EC powder was obtained from the National Marine Biodiversity Institute of Korea. EC powder underwent a sequential extraction process. After sonication, the powder was immersed in 70% ethanol for 4 to 6 h at 37 °C, protected from light. The ethanol extract was concentrated using a rotary evaporator, and the remaining aqueous solution was freeze-dried to obtain the powdered ethanol (EtOH) extract. Subsequently, the remaining solid was successively extracted with ethyl acetate, butanol, and water under the same conditions. After each extraction, the organic solvents were evaporated using a rotary evaporator, while the water extract was freeze-dried to obtain the powdered forms of ethyl acetate (ETAC), butanol, and water (H_2_O) extracts. Multiple batches of the EC powder were tested to confirm consistency.

The 3T3-L1 preadipocytes were purchased from the American Type Culture Collection (ATCC, Manassas, VA, USA). Dulbecco’s modified Eagle’s medium (DMEM), bovine calf serum (BCS), fetal bovine serum (FBS), penicillin–streptomycin (P/S), insulin, and trypsin-EDTA were purchased from Gibco (Gaithersburg, MD, USA). Zinc protoporphyrin (ZnPP), 3-isobutyl-1-methylxanthine (IBMX), dexamethasone (DXM), and insulin were purchased from Sigma-Aldrich Co. (St. Louis, MO, USA). Primary antibodies for PPARγ, SREBP-1, C/EBPα, aP2, β-actin, LPL, FAS, p-HSL, HSL, HO-1, Nrf2, Lamin-B, AMPK, PGC1α, and UCP1 were purchased from Santa Cruz Biotechnology (Santa Cruz, CA, USA), and phospho-HSL Ser660 and p-AMPKα from Abcam (Cambridge, UK).

### 4.2. Identification of Compounds

The identification of compounds was performed using an ultra-high-resolution Q-TOF LC MS/MS system (maXis-HD™, Bruker Daltonics, Bremen, Germany). The extracts were reconstituted in methanol at a concentration of 1 mg/mL, and a 10 µL aliquot of each reconstituted sample was filtered through a 0.22 µm syringe filter to remove particulates. For chromatographic separation, 10 µL of the filtered sample was injected into the LC system utilizing an Acquity UPLC BEH C18 column (2.1 mm × 50 mm, 1.7 µm particle size). The mobile phase consisted of water with 0.1% formic acid (solvent A) and acetonitrile with 0.1% formic acid (solvent B). The gradient elution program was set as follows: 0–1 min at 5% B, 1–10 min from 5% to 95% B, 10–12 min at 95% B, 12–13 min from 95% to 5% B, and 13–15 min at 5% B for re-equilibration. The flow rate was 0.3 mL/min, and the column temperature was maintained at 40 °C.

The Q-TOF MS was operated in positive ion mode, with source parameters set as follows: capillary voltage at 4.5 kV, end plate offset at −500 V, nebulizer pressure at 2.0 bar, dry gas flow rate at 8 L/min, and dry gas temperature at 200 °C. Data acquisition covered a mass range of *m*/*z* 50–1500. MS/MS experiments were conducted using collision-induced dissociation (CID) with nitrogen as the collision gas at a pressure of 1.5 bar. The acquired data were processed using Biotools 3.2 (Bruker Daltonics, Bremen, Germany) and compound identification was achieved by comparing the MS/MS spectra with known reference standards and databases such as METLIN and MassBank.

### 4.3. Cell Culture and Cell Viability

The 3T3-L1 preadipocytes were procured from ATCC (Manassas, VA, USA), cultured in DMEM supplemented with 10% BCS and 1% penicillin/streptomycin, and maintained at 37 °C in a 5% CO_2_ incubator. Cell viability was assessed using the MTT assay. The cells were treated with different concentrations of EC extract (10, 50, 100 μg/mL) for 24 h. A final solution of 5 mg/mL MTT was added to each well, and the cells were incubated at 37 °C for an additional 4 h. The MTT-containing media was then gently removed, and 100 μL of DMSO was added to each well to dissolve the intracellular formazan crystals. The absorbance was measured at 540 nm using a microplate reader (Multiskan™ GO, Thermo Scientific™, Waltham, MA, USA).

### 4.4. Cell Differentiation and Oil Red O Staining

To induce adipogenesis, cells were incubated in DMEM supplemented with 10% FBS, 0.5 mM IBMX, 1 μM dexamethasone, and 4 μg/mL insulin for 2 days. The adipocyte culture medium, consisting of DMEM with 10% FBS and 4 μg/mL insulin, was refreshed every 2 days. For staining, cells were washed twice with PBS and fixed with 10% formalin for 1 h at room temperature. Subsequently, Oil Red O solution was added and allowed to stain the cells for 10 min. Excess stain was removed by rinsing twice with distilled water. Images of the 3T3-L1 adipocytes were captured using an inverted microscope (DMI6000, Leica, Wetzlar, Germany). The stain was then extracted with isopropanol and quantified by measuring absorbance at 510 nm using a microplate reader (Multiskan™ GO).

### 4.5. Medium Biochemistry

The 3T3-L1 adipocytes were harvested, washed with PBS, and lysed. The lysates were centrifuged at 12,000× *g* for 10 min at 4 °C to remove debris. Total cholesterol and triglyceride levels were measured using assay kits (Biomax, Seoul, Republic of Korea) as described in previous studies [46]. Free glycerol levels were determined using free glycerol reagent (F6428, Sigma-Aldrich, St. Louis, MO, USA). For quantification of pro-inflammatory cytokines in serum, ELISA kits from Biolegend (San Diego, CA, USA) were used according to the manufacturer’s instructions.

### 4.6. HO-1 Inhibition

To evaluate the role of HO-1, certain groups of 3T3-L1 preadipocytes were pre-treated with ZnPP, at a concentration of 5 µM for 1 h prior to the addition of the samples. Subsequently, adipogenesis was induced following the same protocol as previously described.

### 4.7. Determination of Intracellular ROS

ROS generation in cells was detected in a 96-well black plate for quantification, following previous research [47]. After 8 days of treatment, cells were incubated with 20 μM DCFH-DA in Hank’s Balanced Salt Solution for 20 min at 37 °C. The intracellular ROS levels were then quantified by measuring fluorescence intensity, with excitation at 485 nm and emission at 528 nm, using an Infinite^®^ 200 Pro microplate reader (TECAN, Männedorf, Switzerland). Fluorescence microscopy images were captured using a DMI6000 microscope (Leica, Wetzlar, Germany).

### 4.8. Western Blot

Cells were lysed in ice-cold RIPA buffer with a protease inhibitor tablet (1 per 20 mL). Lysates were centrifuged at 12,000 rpm for 20 min at 4 °C, and the supernatant was collected. Protein content was measured using a BCA assay. Equal protein amounts were mixed with loading buffer, separated by gel electrophoresis, and transferred to a nitrocellulose membrane. The membrane was blocked with 5% skim milk, incubated with primary antibodies overnight at 4 °C, and then with secondary antibodies for 2 h at room temperature. Protein detection was performed using a chemiluminescence ECL kit, and images were captured with a Davinch-Chemi Imager™ (CAS400SM, Core Bio, Seoul, Republic of Korea). Densitometry analysis was performed using ImageJ software (ver.1.54g).

### 4.9. Data Analysis

Statistical analysis was conducted using SigmaPlot^®^ 12.0 (Systat Software Inc., San Jose, CA, USA). Data from all experiments are presented as mean ± standard deviation (SD). A Student’s *t*-test was applied to evaluate statistical significance, with a threshold set at *p* < 0.05.

## 5. Conclusions

The findings highlight the HO-1/Nrf2 pathway’s pivotal role in EC-ETAC’s anti-obesity effects. EC-ETAC modulates this pathway to inhibit adipogenesis, promote lipolysis, and enhance adipose browning. Inhibiting HO-1 diminishes these effects, underscoring its importance in adipocyte metabolism and obesity treatment. Further research on the HO-1/Nrf2 axis could reveal new anti-obesity strategies. The potential of EC-ETAC to activate this pathway and induce adipose browning suggests significant therapeutic potential, warranting further investigation.

## Figures and Tables

**Table 1:** Compounds detected in *Ecklonia cava* ethyl acetate extract.

Component Name	Observed *m*/*z*	Mass Error (ppm)	Observed RT (min)	Area (%)
Dieckol	741.0727	−0.8	12.27	21.49%
Dibenzodioxin-fucodiphloroethol	743.0882	−1	10.32	11.33%
8,8′-Bieckol	741.0726	−1	9.29	10.20%
2-O-(2,4,6-Trihydroxyphenyl)-6,6′-Bieckol	865.0885	−1.1	9.38	8.53%
7-Phloroeckol	495.0570	0.2	8.53	7.48%
Phlorofucofuroeckol A	601.0615	−1.4	14.56	6.72%
Fucodiphlorethol G	497.0729	0.7	2.76	5.76%
Fucodiphlorethol G Isomer	497.0727	0.4	3.4	5.00%
2-O-(2,4,6-Trihydroxyphenyl)-6,6′-Bieckol Isomer	865.0880	−1.6	9.01	5.00%
Dibenzodioxin-fucodiphloroethol Isomer	743.0876	−1.8	8.84	4.84%
7-Phloroeckol Isomer	495.0572	0.6	8.66	3.34%
2-[2-(3,5-Dihydroxyphenoxy)-3,5-dihydroxyphenoxy]-1,3,5-Benzenetriol Isomer	373.0565	−0.1	3.7	2.82%
2-[2-(3,5-Dihydroxyphenoxy)-3,5-dihydroxyphenoxy]-1,3,5-Benzenetriol	373.0565	0	3.18	2.38%
6,6′-Bieckol	741.0723	−1.4	11.81	1.69%
3,7-Bis [3,5-dihydroxy-4-(2,4,6-trihydroxyphenoxy)phenoxy]dibenzo[b,d]furan-2,4,6,8-tetrol	759.0826	−1.7	7.5	0.75%
Spinatoside	521.0935	−0.4	4.44	0.66%
5,6-Dihydroxy-4-oxo-2-(3,4,5-trihydroxyphenyl)-4H-chromen-7-yl 6-O-acetyl-beta-D-glucopyranoside	521.0936	−0.1	5.11	0.61%
2-O-(2,4,6-Trihydroxyphenyl)-6,6′-Bieckol Isomer 2	865.0880	−1.6	10.05	0.54%

## Data Availability

The data presented in this study are available on request from the corresponding authors.
